# Heterogeneity in Reported Outcome Measures after Surgery in Superior Canal Dehiscence Syndrome—A Systematic Literature Review

**DOI:** 10.3389/fneur.2017.00347

**Published:** 2017-07-24

**Authors:** Mira E. Ossen, Robert Stokroos, Herman Kingma, Joost van Tongeren, Vincent Van Rompaey, Yasin Temel, Raymond van de Berg

**Affiliations:** ^1^Faculty of Medicine, Maastricht University, Maastricht, Netherlands; ^2^Department of Otorhinolaryngology and Head & Neck Surgery, Maastricht University Medical Center, Maastricht, Netherlands; ^3^Faculty of Physics, Tomsk State University, Tomsk Russian Federation, Tomsk, Russia; ^4^Faculty of Medicine and Health Sciences, University of Antwerp, Antwerp, Belgium; ^5^Department of Otorhinolaryngology and Head & Neck Surgery, Antwerp University Hospital, Edegem, Belgium; ^6^Department of Neurosurgery, Maastricht University Medical Center, Maastricht, Netherlands

**Keywords:** superior canal dehiscence syndrome, middle fossa, transmastoid, plugging, resurfacing

## Abstract

**Background:**

Superior canal dehiscence syndrome (SCDS) can be treated surgically in patients with incapacitating symptoms. However, the ideal treatment has not been determined.

**Objectives:**

This systematic literature review aims to assess available evidence on the comparative effectiveness and risks of different surgical treatments regarding: (1) symptom improvement; (2) objectively measurable auditory and vestibular function; (3) adverse effects, and (4) length of hospitalization.

**Search method and data sources:**

A systematic database search according to PRISMA statement was conducted on Pubmed, Embase, and Cochrane library. In addition, reference lists were searched. No correspondence with the authors was established. The last search was conducted on June 9, 2017.

**Study eligibility criteria:**

Retrospective and prospective cohort studies were held applicable under the condition that they investigated the association between a surgical treatment method and the relief of vestibular and/or auditory symptoms. Only studies including quantitative assessment of the pre- to postoperative success rate of a surgical treatment method were included. Case reports, reviews, meta-analysis, and studies not published in English, Dutch, or German were excluded.

**Data collection and analysis:**

The first author searched literature and extracted data; the first and last analyzed the data.

**Main results:**

Seventeen studies (354 participants, 367 dehiscences) met the eligibility criteria and were grouped according to surgical approach. Seven combinations of surgical approaches and methods for addressing the dehiscence were identified: plugging, resurfacing, or a combination of both through the middle fossa (middle fossa approach); plugging, resurfacing, or a combination of both through the mastoid (transmastoid approach); round window reinforcement through the ear canal (transcanal approach). Several studies showed high internal validity, but quality was often downgraded due to study design (1). Outcome measures and timing of postsurgical assessment varied among studies, making it unfeasible to pool data to perform a meta-analysis.

**Conclusion:**

A standardized protocol including outcome measures and timeframes is needed to compare the effectiveness and safety SCDS treatments. It should include symptom severity assessments and changes in vestibular and auditory function before and after treatment.

## Introduction

### Rationale

#### Reason for Conducting This Research

Superior canal dehiscence syndrome (SCDS) is a rare condition in which a hole in the superior semicircular canal causes sound and pressure waves to evoke vestibular and auditory symptoms. Symptoms can include sound- or pressure-induced vertigo (Tullio phenomenon), autophony, pulsatile tinnitus, bone conduction (BC) hyperacusis, conductive hearing loss, and “brain fog” (2–5). This broad variety of symptoms can make it difficult to distinguish SCDS from other neurological and otological pathologies. Migraine headaches for example can cause similar symptoms or present concurrently, but are treated differently (6). Patients with SCDS may be treated conservatively with education and observation. In more severe cases, however, surgical treatment is warranted when symptoms become incapacitating. Since the discovery of SCDS in 1998 by Lloyd Minor, who first referred to it as the “third window effect,” a number of surgical techniques have been described (3, 5). Most techniques aim to relieve the patient’s symptoms by closing the dehiscence and/or the plugging the semicircular canal. A recent alternative approach has suggested that occluding the round window through the ear canal may partially dampen the “third mobile window” effect (7). The dehiscence can be approached *via* middle cranial fossa of the skull (“middle fossa approach”) or *via* the mastoid (“transmastoid approach”). Both middle fossa and transmastoid approaches allow resurfacing of the dehiscence as well as plugging of the canal and different materials have been used including fascia, bone chips, bone wax, fibrin glue and bone dust. Both approaches have shown high success rates in terms of symptom relief (5); however, each carries risks.

The ideal treatment should combine the safest approach with the most effective and durable closure technique. This ideal treatment has yet to be determined. A number of reviews have aimed to address this issue but have been limited to comparing highly variable outcome measures used to assess the different techniques (8–10). Despite an excellent internal setup, the risk of bias in most published studies is relatively high due to their retrospective study design and small sample size. Furthermore, different outcome measures have been used among the studies. Therefore, results can strictly speaking only be compared qualitatively and not be pooled. For instance, when comparing hearing outcomes across groups, studies report different frequency ranges (e.g., 0.25–4 vs. 0.25–8 kHz). A high-frequency hearing loss may therefore be reported in one study, but not in another that only reports lower frequencies. Since systematic reviews provide clinical overviews and also advise clinicians on best practice methods, conclusions by authors should be drawn with care and be based on properly analyzed, pooled data. The PRISMA statement was chosen as a guideline as it focuses particularly on the evaluation of interventions. It is the preferred reporting guideline for systematic reviews and meta-analyses (11).

### Objective

This systematic review aims to assess the evidence on the comparative efficacy of the different surgical treatments with respect to: (1) alleviating symptoms (BC hyperacusis, tinnitus, autophony, hearing, aural fullness, noise- and pressure-induced vertigo, and general disequilibrium); (2) change in objectively measurable auditory and vestibular function; (3) presence of adverse effects, and (4) length of hospitalization.

## Methods

### Protocol

To structure this systematic review, the PRISMA statement for reporting systematic reviews was applied.

### Eligibility Criteria

#### Inclusion Criteria

Retrospective and prospective cohort studies were included if they analyzed the association between at least one surgical treatment method and its effect on symptom relief or auditory and vestibular function. To guarantee a minimum level of comparability, the success rates had to be assessed in a quantitative manner and had to be based on pre- and postoperative data.

In the context of this review, it was not feasible to translate literature, therefore only literature published in English, Dutch or German (i.e., languages spoken by the reviewing authors) was included. The search was constrained to studies on humans and to literature that was accessible through the library services of Maastricht University, the Academic Hospital Maastricht, or the Dutch Inter Library Loan Service.

#### Exclusion Criteria

Studies were excluded if they did not analyze success rates in a quantitative manner, did not assess outcomes of one particular surgical treatment method, or did not use symptom relief as an outcome measure. Furthermore, the following types of literature were excluded: case reports, reviews, meta-analyses, and studies not published in English, Dutch, or German.

### Information Sources

A systematic search was performed in the PubMed database, Embase database, and Cochrane library of clinical trials. Furthermore, the reference lists of selected articles were screened for additional literature. No unpublished literature was integrated into this review. The last search was conducted on June 9, 2017.

### Search

We searched all three databases for abstracts, titles and key words containing suitable search terms. While searching PubMed we used the following terms:

((superior canal dehiscence syndrome) AND (((((((((((“Tinnitus”[Mesh]) OR tinnitus)) OR ((“Hyperacusis”[Mesh]) OR sound sensitivity)) OR autophony) OR phonophobia) OR ((“Hearing Loss”[Mesh]) OR hearing impairment)) OR aural fullness) OR ((“Pressure”[Mesh]) OR pressure)) OR ((“Vertigo”[Mesh]) OR imbalance)) OR oscillopsia)) AND ((((((middle fossa approach) OR transmastoid approach) OR endaural approach) OR resurfacing) OR plugging) OR ((“Surgical Procedures, Operative”[Mesh]) OR surgical treatment))

Embase Database was searched using the following search terms:

#1 “superior canal dehiscence syndrome”
#2 “tinnitus” or “hyperacusis” or “sound sensitivity” or “autophony” or “phonophobia” or “hearing loss” or “hearing impairment” or “oral fullness” or “pressure” or “vertigo” or “imbalance” or “oscollopsia” or “tullio phenomenon”
#3 “surgical treatment” or “surgical procedure” or “operation” or “middle fossa” or “middle fossa approach” or “transmastoid” or “transmastoid approach” or “endaural approach” or “resurfacing” or “plugging”

Cochrane Database of Clinical Trials was consequently searched with equivalent terms:

#1 “superior canal dehiscence”: ti,ab,kw or “superior canal dehiscence syndrome”: ti,ab,kw or “MeSH descriptor: [Semicircular Canals] explode all trees” 0
#2 “MeSH descriptor: [Tinnitus] explode all trees” or “MeSH descriptor: [Hyperacusis] explode all trees” or “MeSH descriptor: [Hearing Loss, Sensorineural] explode all trees” or “aural fullness”: ti,ab,kw or “MeSH descriptor: [Pressure] explode all trees” or “vertigo”: ti,ab,kw or “Tullio’s phenomenon”: ti,ab,kw or “autophony”: ti,ab,kw 6317
#3 “surgical treatment”: ti,ab,kw or “MeSH descriptor: [General Surgery] explode all trees” or “operation”: ti,ab,kw 24360
#4 “middle fossa”: ti,ab,kw or “resurfacing”: ti,ab,kw or “endaural approach”: ti,ab,kw or “plugging”: ti,ab,kw 25289
#5 #3 or “middle fossa”: ti,ab,kw or “resurfacing”: ti,ab,kw or “endaural approach”: ti,ab,kw or “plugging”: ti,ab,kw 25289
#6 #1 and #2 and #5 0
#5 #2 and #5 234

### Study Selection and Data Collection Process

The first author screened titles and abstracts of the found articles to evaluate whether the content fit the inclusion and exclusion criteria. The first and last author analyzed the data in consensus.

### Data Items

Data elements extracted from each study were as follows: study design, setting and duration of follow-up, country of origin, the number of participants, number of participants lost during follow-up, assessment tools and outcome measures.

### Risk of Bias in Individual Studies

To evaluate the risk of bias for each study, specific methodological criteria were applied.

If relevant methodology was not addressed in the article, it was considered a risk for bias. The elements assessed for risk of bias that were included are presented in Table 1. A three-point scale was used to estimate the risk of bias for each component. The plus sign (+) indicates a low risk of bias. The sign (+/−) is associated with elements that were rated with a moderate or unknown risk of bias. Studies were marked with a minus (–) if there was a high possibility of bias.

**Table 1:** Risk of bias per study.

Study ID	In- and exclusion criteria	Selection bias	Participant characteristics	Blinding of participants, personnel	Reproducibility (techniques well described)	Correction for confounding	Follow-up	Quality of outcome measure	Loss to follow-up	Information bias	Conflict of interest	Selective reporting of outcome
Remenschneider et al. (16)	+	−	+	−	+	−	+	+	+	−	+	+/−
Chung et al. (30)	+/−	−	+	−	+	−	+/−	−	+	−	+	+
Thomeer et al. (19)	+	−	+	−	+	−	+	+	+	−	+/−	±
Goddard and Wilkinson (20)	+	−	+/−	−	+	−	+	+	+	−	+/−	±
Ward et al. (34)	+	−	+/−	−	+	−	+/−	+	+	−	+/−	±
Agrawal et al. (26)	+	−	+/−	−	+	−	+/−	+	+	−	+/−	±
Crane et al. (18)	+	−	+	−	+	−	+	+	+	−	+/−	±
Crane et al. (21)	+	−	+	−	+	−	+	+	+	−	+/−	±
Carey et al. (14)	+	+/−	±	−	+	−	+/−	+	−	+/−	+	+
Phillips et al. (22)	+	−	+	−	+	−	+	+	+	−	+/−	+
Limb et al. (23)	+	−	−	−	+	−	+/−	+	+	−	+/−	+
Hillman et al. (24)	+	−	+	−	+	−	+	−	+	−	+/−	−
Van Haesendonck et al. (10)	+	−	+	−	+	−	+/−	+	+	−	+/−	−
Beyea et al. (29)	+	−	+/−	−	+	−	+/−	+	+	−	+	−
Agrawal and Parnes (12)	+	−	+/−	−	+	−	+/−	+	+	−	+	+/−
Fiorino et al. (25)	+	−	+	−	+	−	+	+/−	+	−	+/−	+
Amoodi et al. (17)	+	−	−	−	+	−	+	+/−	+	−	+/−	±
Lundy et al. (15)	+	−	+/−	−	+	−	+	+	+	−	+/−	+
Silverstein et al. (7)	+	−	+/−	−	−/+	−	+/−	±	+	−	+/−	+

### Summary of Outcome Measures

Different assessment tools were used (Table 2). Outcome measures could be divided into subjective and objective measures. Every measure that evaluated the subjective symptom improvement in patients was considered a primary outcome measure. Subjective outcomes were most-commonly extracted from non-standardized pre- or postoperative interviews and questionnaires. However, some studies used validated measures such as the dizziness handicap inventory (DHI), autophony index (AI), Hearing Handicap Inventory (HHI), or health utility value (HUV). The latter is an assessment tool to calculate the quality-adjusted life years, representing quality of life.

**Table 2:** Assessment tools for subjective and objective outcome measures.

Technique		Study	Assessment subjective symptoms	Assessment objective measurements					Time of measurement post surgery	Adverse effects
				Audiometrics	Vestibular-evoked myogenic potentials (VEMPs)	Head impulse test	Calorics	Induced nystagmus		
Middle fossa approach	P	Remenschneider et al. (16) (*n* = 14)	Health utility value, autophony index (AI), dizziness handicap inventory (DHI), and Hearing Handicap Inventory	x	x	x	x	x	3 months	x
	
	P + R	Chung et al. (30); 21 ears (*n* = 18)	Anamnesis	x	x	x	x	x	x	x
		
		Thomeer et al. (19); 16 ears (*n* = 13)	Anamnesis	*Tone*: air conduction (AC), bone conduction (BC), air bone gap (ABG): 0.25–1.0 and 0.5–4.0 kHz	Cervical vestibular-evoked myogenic potentials (cVEMPs): threshold, amplitude	x	x	x	D7 and 1 month	Reported
		
				*Speech*: speech reception threshold						
		
		Goddard and Wilkinson (20); 24 ears (*n* = 23)	Anamnesis	*Tone*: AC, BC: 0.5–3 kHz, ABG: 0.5–3 kHz	x	x	x	x	x	Reported
		
				*Speech*: Word Recognition Score						
		
		Ward et al. (34); 43 ears (*n* = 40)	x	*Tone*: AC: 0.25–8 kHz, BC: 0.25–4 kHz, ABG: 0.25–2 kHz	x	x	x	x	x	Reported
		
		Agrawal et al. (26); 42 ears	x	x	x	1. Clinical	x	x	x	x
		
						2. Search coil: horizontal canals				
		
		Crane et al. (18); 19 ears	AI, DHI	*Tone*: AC: 0.25–8 kHz, BC: 0.25–4 kHz, ABG: 0.25–4 kHz	cVEMPs: thresholds	x	x	x	3 months: AI, DHI	Reported
		
		Crane et al. (21); 19 ears	DHI	*Tone*: AC 0.25–8 kHz, BC: 0.25–4 kHz	cVEMPs: thresholds, auditory stimuli	x	x	x	3 months: DHI	x
		
		Carey et al. (14); 19 ears	Anamnesis	x	x	Search coil: all canals	x	x	1.5–7 months	Reported
		
		Phillips et al. (22); 5 ears	Anamnesis	*Tone*: AC, BC (frequencies not announced)	cVEMPs: thresholds	x	x	x	4 months	Reported
		
				*Speech*: perception						
		Limb et al. (23); 29 ears	x	*Tone*: AC: 0.25–8 kHz, BC: 0.5–4 kHz, ABG: 0.25–8 kHz	x	x	x	x	x	x
	
				*Speech*: SDS						
	R	Hillman et al. (24); 16 ears (*n* = 13)	Anamnesis	*Tone*: AC, BC: 0.5–3 kHz, ABG (frequencies not announced)	x	x	x	x	>3 months	x
				*Speech*: SDS						

Transmastoid approach	P	Van Haesendonck et al. (10); 12 ears	Anamnesis	*Tone*: AC, BC: 0.5–4 kHz, ABG (frequencies not announced)	x	x	x	x	x	Reported
		Beyea et al. (29); 16 ears	Anamnesis	x	x	x	x	x	x	Reported
		
		Agrawal and Parnes (12); 3 ears	Anamnesis	*Tone*: AC, BC, ABG (frequencies not announced)	x	x	x	x	x	Reported
	
	P + R	Fiorino et al. (25); 6 ears	Anamnesis	*Tone*: AC, BC (frequencies not announced)	VEMPs: thresholds	x	Procedure not described	Sound and pressure induced, not described.	1.5–2 months	Reported
	
	R	Amoodi et al. (17); 4 ears	Anamnesis	*Tone*: AC, BC: 0.25–8 kHz	x	x	x	Sound and pressure induced, procedure not described.	Not reported, follow-up varied between 1.5 and 4 years	Reported
		Lundy et al. (15); 37 ears	Scale from “worsening” to “much better”	x	x	x	x	x	>3 months	x

Transcanal approach	RW	Silverstein et al. (7); 22 ears	Superior canal dehiscence syndrome questionnaire	x	x	x	x	x	x	x
			(7-point Likert type scale)							

Measures that evaluated auditory or vestibular function were considered secondary outcome measurements in this review. The following objective outcome measures considered valid: standard pure-tone audiometry, word recognition/speech discrimination score, speech reception threshold (SRT), cervical vestibular-evoked myogenic potential (cVEMP), caloric testing, rotational chair testing, and electronystagmography.

## Results

### Selected Studies

The query returned 67 articles in PubMed, 20 articles in the Embase database, and 4 articles in the Cochrane Library. After removing duplicates 65 titles and abstracts were evaluated and 44 were excluded due to study design (case reports and reviews), outcome measures or because they were not available in the required languages. Reference lists of the remaining 21 articles were screened by title and abstract, resulting in an additional 7 articles for further evaluation. These 28 eligible studies were further analyzed for conformity with the remaining inclusion and exclusion criteria. After a full text screening, eight studies were excluded because they did not contain a quantitative measure of success. In addition, one article was excluded because it did not report the surgical approach. Nineteen studies were therefore included in this review.

### Study Characteristics

#### Study Design

The majority (18) of studies were retrospective chart reviews. The patient data collected for these chart reviews all contained pre- and postoperative information. However, the postoperative follow-up varied between 1 and 7 years. Only one prospective trial met the inclusion criteria.

#### Country

All 19 studies were conducted in countries with a mainly Caucasian population, either originating from the USA, Canada, France, Belgium, Italy, or Australia.

#### Sample Size

The number of cases in the retrospective studies varied between 3 (12) and 43 (13). The single prospective study consisted of 19 participants (14).

#### Setting

The majority of studies (18) were set in single, tertiary referral otology centers. There was only one multi-center retrospective chart review that evaluated patients from four institutions (7).

#### Diagnosis

In every study, the diagnosis of SCDS was based on vestibular and auditory symptoms and evidence of a dehiscence on CT imaging. In the majority of studies, VEMP thresholds were also used as a diagnostic tool but not uniformly and in many studies they were not mandatory for diagnosis. Clinically, patients mainly suffered from a combination of symptoms such as sound- and pressure-induced vertigo, chronic disequilibrium, autophony, aural fullness, pulsatile tinnitus, and hyperacusis. However, not all studies reported eligibility criteria for surgery and in some cases patients underwent surgery while suffering from one symptom alone (10). As mentioned above, migraine headaches are an important consideration in the differential diagnosis. However, it was not explicitly stated in any article whether migraine headaches were ruled out as the cause of the symptoms.

#### Approach and Closing Technique

As mentioned above there were two primary surgical approaches (middle cranial fossa and transmastoid) and three different reparative techniques (plugging or resurfacing the superior semicircular canal alone and resurfacing with canal plugging). An alternative was transcanal reinforcement of the round window. Twelve studies examined the success rates of the middle cranial fossa approach. In 1 of the 12 studies, the superior semicircular canal was plugged, in 10 studies, the canal was plugged and resurfaced, and 1 study described the middle cranial fossa approach with resurfacing of the canal. The success rates for the transmastoid approach were assessed in six studies. Of the six studies, three involved plugging, one involved a combination of plugging and resurfacing, and two involved resurfacing alone [i.e., “cartilage cap occlusion” (15)]. Reinforcement of the round window through the ear canal was assessed in one study. It should be mentioned that one of the studies that mainly assessed plugging *via* the middle fossa also contained two patients who underwent transmastoid plugging (16). These cases could not be included in the analyses of this review since pre- and postoperative data were not reported in detail.

#### Follow-up

The follow-up of the retrospective studies, meaning the time until the last recorded data, varied from 1 month (13) to 4 years (17). A total of six studies, including the previously mentioned prospective study, did not report the duration of follow-up.

#### Loss to Follow-up

No loss to follow-up was reported in any retrospective study. In the prospective study, a loss to follow-up of 13 patients was reported resulting in a final population of 19 patients (14).

#### Source of Data

Retrospective studies collected and evaluated existing pre-and postoperative data from medical records. The one prospective study compared the existing preoperative data with new postoperative clinical records and auditory and vestibular measurements.

### Risk of Bias within Studies

#### Inclusion and Exclusion Criteria

All studies listed the inclusion and exclusion criteria.

#### Selection Bias

All retrospective studies were prone to selection bias and were therefore all graded with a (−). The prospective study was graded with a (+/−) due to an unclear risk of bias due to a lack of information on the patient selection process.

#### Participant Characteristics

With the exception of two studies, the patients’ symptoms were clearly described. However, other patient demographics like age, sex, comorbidities, or otological history were often not described.

#### Blinding

Since blinding is not applicable to retrospective studies, 18 of the articles were graded with a minus (−). In the prospective study, the blinding of patients or surgeons was not required since there was no alternative treatment and therefore no control group.

#### Reproducibility

The surgical technique was well described in all 19 studies. However, in one study, surgeons used different techniques and materials, limiting reproducibility (7).

#### Correction for Confounding

Correction for confounding was not performed in any study.

#### Follow-up

The follow-up time differed greatly among studies. However, all studies had similar definitions for short-term and intermediate-term postoperative stages. This made it possible to compare early vs. late stage outcomes across the different studies.

Judgment on the length of follow-up depended upon the research question of each study. If a study aimed to investigate the short-term outcomes of the operation, several days could be considered a sufficient follow-up time. In comparison, a study that explored long-term outcomes required a follow-up of at least 12 months.

Out of the 19 studies, 10 presented a follow-up period of more than 3 months and were graded with a (+). We considered a follow-up of more than 3 months as sufficient since the immediate postoperative side effect should, by that time, be resolved. The remaining nine studies presented a follow-up time that was reasonably aligned with their research question (e.g., short-term follow-up when focusing on immediate effects) and were therefore graded with a (+/−).

#### Quality of Outcome Measure

The overall quality of outcome measures was moderate to low for the primary outcome measures that assessed subjective symptom improvement. They were mainly assessed through anamnesis and not standardized questionnaires that, for example, described “improvement,” “worsening,” or “no change in symptoms” (15). Standardized rating scales were: a questionnaire to measure an AI (18), the DHI, the HHI, the HUV, and a non-validated 7-point Likert type scale to assess the severity of each SCDS symptom (7).

The overall quality of outcome measure for the secondary objective outcome measurements was high. Theses included the following: audiometry (10, 12, 13, 15, 17–25), vestibular-evoked myogenic potentials (VEMPs) (18, 19, 21, 22, 25), head impulse test (HIT) (14, 26), video head impulse test (vHIT) (26), caloric tests (25), and presence of induced nystagmus (17, 25). Audiometry comprised the air conduction (AC) pure-tone average (PTA), the BC PTA, the air bone gap (ABG) closure, and in some cases the SRT or the Word Recognition Score (WRS). A number of studies reported that values were calculated according to American Academy of Otolaryngology-Head and Neck Surgery Foundation (AAO-HNS) reporting guidelines (27). However, in the other cases, the precise assessment of outcome measures was not described. An additional outcome measure was the length of hospital stay in days and the presence of adverse effects.

#### Loss to Follow-up

All 18 retrospective reports were graded with a (+). The prospective study was considered to have a high risk of bias and was graded with a (−).

#### Information Bias

In a retrospective context, it was not possible to determine whether the information being used for the study was faulty or inaccurate. The risk for information bias was therefore considered high in all retrospective studies. All 18 retrospective chart reviews were therefore graded with a (−). Regarding the one prospective study, the risk for information bias was unclear and was graded with a (+/−).

#### Conflict of Interest

No conflicts of interest were reported, and there was no reason to suspect such in any of the studies. All studies were marked with a (+).

#### Selective Reporting of Outcome

Three articles were inconsistent with the reported numbers and results. They were therefore considered to have a high risk for bias and were therefore graded with a (−).

### Results of Individual Studies per Outcome Measure

To increase comparability, the level of evidence and level of recommendation was added to each conclusion that follows a result analysis (28).

#### Primary Outcomes—Subjective Improvement (Table 3)

**Table 3:** Subjective improvement in affected ears.

		Study	Improvement of symptoms in affected ears									
			HA	T	AP	H	AF	NIV	PIV	GD	Headache	Quality of life
Middle fossa	P	Remenschneider et al. (16) (*n* = 14)	x	x	*Mean autophony index (AI) (n *=* 14)*: Pre: 32.7 (28.0) Post: 4.8 (8.3) *p* < 0.001	*Mean Hearing Handicap Inventory (n *=* 14)*: Pre: 41.8 (27.9) Post: 26.7 (30.2) *p* = 0.140	x	x	x	*Mean dizziness handicap inventory (DHI) (n *=* 14)*: Pre: 48.7 (23.4) Post: 38.2 (30.7) *p* = 0.200	x	*Mean health utility value (SD)* Pre: 0.65 (0.12) Post: 0.79 (0.12) *p* < 0.001
	
	P + R	Chung et al. (30); 21 ears (*n* = 18)	x	Pre: 12/18 Post: 3/18 Developed: 1/3 *p* = 0.0059	Pre: 16/18 Post: 5/18 *p* = 0.0005	Pre: 10/18 Post: 4/10 *p* = 0.0858	Pre: 7/18 Post: 3/18 Developed: 2/3 *p* = 0.2642	Pre: 13/18 Post: 6/18 Developed: 1/6 *p* = 0.0437	Pre: 13/18 Post: 6/18 Developed: 1/6 *p* = 0.0437	Pre: 11/18 Post: 5/18 Developed: 2/5 *p* = 0.0922	Pre: 5/18 Post: 4/18 Developed: 1/4 *p* = 1.0000	x
		
		Thomeer et al. (19); 16 ears (*n* = 13)	x	Resolved: Pulsatile: 12/12 Non-pulsatile: 0/1	Resolved: 9/9	x	Resolved: 6/6	Resolved: 14/16	Resolved: 10/10	Resolved: 14/16	x	x
		
		Goddard and Wilkinson (20); 24 ears (*n* = 23)	x	x	Resolved: 12/15	x	Resolved: 16/20	Resolved: 8/8	Resolved: 8/11	Resolved: 16/24	x	x
		
		Ward et al. (34); 43 ears (*n* = 40)	x	x	x	x	x	x	x	x	x	x
		
		Agrawal et al. (26); 42 ears	x	x	x	x	x	x	x	x	x	x
		
		Crane et al. (18); 19 ears			*Mean AI (n *=* 18)*: Pre: 42 ± 27 (range 0–86) Post: 9 ± 22 (range 0–82) *Mean change*: 33 (*p* < 0.01)					*Mean DHI (n *=* 18)*: Pre: 48.22 Post: 27.77	x	x
		
		Crane et al. (21); 19 ears	x	x	x	x	x	x	x	*Mean DHI (n *=* 19)*: Pre: 44 ± 24 Post: 18 ± 15 *p* < 0.01 *Mean ↓*: 26 ± 25 *p* < 0.01 Decreased: 17/19 Increased: 2/19	x	x
		
		Carey et al. (14); 19 ears	x	x	x	x	x	Resolved: 19/19	Resolved: 19/19	x	x	x
		
		Phillips et al. (22); 5 ears			Improved: 5/5					Transient increase: 4/5 Improved: 5/5	x	x
		
		Limb et al. (23); 29 ears	x	x	x	x	x	x	x	x	x	x
	
	R	Hillman et al. (24); 16 ears (*n* = 13)	x	Resolved: Pulsatile: 2/3 Non-pulsatile: –	x	x	x	x	Resolved: 12/12	Resolved: 12/13	x	x

Transmastoid	P	Van Haesendonck et al. (10); 12 ears	Resolved: 6/8	Resolved: Pulsatile: 6/9 Non-pulsatile: –	Resolved: 11/12	x	x	Resolved: 4/5	Resolved: 2/5 Developed: 1/5	x	x	x
		
		Beyea et al. (29); 16 ears	Resolved: 1/1	Resolved: Pulsatile: 10/10 Non-pulsatile: –	x	Improved: 2/16 Preserved: 14/16	x	x	x	Transient increase: 16/16	x	x
		
		Agrawal and Parnes (12); 3 ears	x	x	Resolved: 1/1	x	x	Resolved: 3/3	Resolved: 1/1	Transient increase: 3/3 Resolved: 3/3	x	x
	
	P + R	Fiorino et al. (25); 6 ears	x	x	x	x	x	Improved: 6/6	Improved: 6/6	Improved: 6/6	x	x
	
	R	Amoodi et al. (17); 4 ears	x	x	x	x	x	x	x	Transient increase: 4/4	x	x
		Lundy et al. (15); 37 ears	x	x	x	x	x	x	x	Questionnaire: Much better: 29 Some better: 5 Same: 2 Worse: 1	x	x

Transcanal	RW	Silverstein et al. (7); 22 ears	x	*7-point Likert scale*: Pulsatile: Pre: 4.6 (SD 2.0) Post: 2.2 (SD 1.4) (1 = not bothered, 7 = disabled) Non-pulsatile: –	*7-point Likert scale*: Autophony: Pre: 4.6 (SD 1.9) Post: 2.2 (SD 1.7) Bone conduction sensitivity: Pre: 4.5 (SD 2.0) Post: 2.0 (SD 1.5)	*7-point Likert scale*: Pre: 2.5 (SD 1.6) Post: 2.2 (SD 1.4)	*7-point Likert scale*: Pre: 4.2 (SD 1.6) Post: 2.5 (SD 1.4)	*7-point Likert scale*: Pre: 5.4 (SD 1.5) Post: 2.3 (SD 1.3)	*7-point Likert scale*: Pre: 3.6 (SD 1.3) Post: 2.0 (SD 1.3)	*7-point Likert scale*: Pre: 4.2 (SD 1.7) Post: 2.1 (SD 1.2)	x	x

##### BC Hyperacusis

Bone conduction hyperacusis was not addressed in the studies that analyzed the effect of resurfacing, or a combination of resurfacing and plugging *via* the middle fossa. However, it was addressed in two studies that investigated transmastoid plugging. Symptoms resolved in seven of the nine affected patients (78%) (10, 29). Improvement and resolution of hyperacusis were also not assessed following round window reinforcement.

Conclusion: Taking these results into account, no comparison could be made among the different approaches; however, BC hyperacusis was often resolved by transmastoid plugging. Grade of evidence: IV; grade of recommendation: D.

##### Pulsatile and Non-Pulsatile Tinnitus

Pulsatile tinnitus was assessed in six studies of which two studies also reported cases of non-pulsatile tinnitus. No evidence was available regarding plugging *via* the middle fossa. In one study plugging plus resurfacing *via* the middle fossa led to a resolution of pulsatile tinnitus in 10/12 (*p* = 0.0059) cases and led to the new development of pulsatile tinnitus in one case (30). In another study, it led to a resolution of pulsatile tinnitus in 12/12 cases (100%) (19). In the same study, one patient experienced non-pulsatile tinnitus before surgery, but it was not reported whether symptoms resolved postoperatively. Resurfacing *via* the same approach led to a resolution in 2/3 cases (67%) (24). Transmastoid plugging resulted in the resolution of 16/19 affected patients (84%) (10, 29). Following round window reinforcement using the transcanal approach, the score for tinnitus dropped from 4.6 to 2.2 on the SCDS 7-point Likert scale, where a score of 1 was not bothersome at all and a score of 7 was extremely bothersome (*n* = 22) (7).

Conclusion: Plugging, resurfacing and a combination of both *via* the middle fossa led to a resolution of symptoms in the majority of cases. This was similar for transmastoid approach plugging. Overall, tinnitus reduced following the transcanal approach to reinforcement of the round window. The middle fossa and transmastoid approach could not be compared with the transcanal approach, because different outcome measures were used. Overall, no validated questionnaires to assess tinnitus were used. Grade of evidence: IV; grade of recommendation: D.

##### Autophony

Plugging *via* the middle fossa showed a significant decrease in AI from 32.7 (SD 28.0) to 4.8 (SD 8.3, *p* < 0.001) (16). Plugging plus resurfacing *via* the middle fossa showed reduction of autophony in four studies, with a resolution of symptoms in 32/40 patients (19, 20, 30) and a significant drop in mean AI Score in 18/19 patients from 42 ± 27 (0–86) to 9 ± 22 with a mean change of 33 points (*p* < 0.01) (18). However, one study reported improvement, but no resolution of autophony symptoms in 5/5 patients (22). No evidence was available on resurfacing alone *via* the middle fossa. Transmastoid plugging led to a resolution in 12/13 patients (10, 12). No evidence was available regarding transmastoid resurfacing. Round window reinforcement led to a significant drop in autophony score from 4.6 to 2.2 on the SCDS 7-point Likert scale (7).

Conclusion: Plugging or plugging and resurfacing the dehiscence using the middle fossa or transmastoid approach as well as round window reinforcement led to significant improvements of autophony symptoms. Comparison of the different techniques was not possible due to different outcome measures. No evidence was available on the effect of resurfacing alone. Grade of evidence: IV; grade of recommendation: D.

##### Subjective Hearing Status

Subjective change in hearing after surgery was addressed in four studies. One study addressed the effect of plugging *via* the middle fossa and indicated a reduction in HHI from 41.8 (SD 27.9) to 26.7 (SD 30.2, *p* = 0.140) (16). Plugging plus resurfacing was also assessed in one study and led to an improvement in hearing in 6/10 cases (*p* = 0.0858) (30). No evidence was available regarding the effects of resurfacing *via* the middle fossa. One study addressed the effect of transmastoid plugging and showed improvement in 2/16 patients and a preservation of hearing in 14/16 patients (29). No evidence was available on the effects of transmastoid resurfacing or a combination of plugging and resurfacing. Reinforcement of the round window also caused no significant change in the SCDS score for hearing on the 7-point Likert scale (7).

Conclusion: Existing evidence showed a non-significant improvement in subjective hearing following middle fossa plugging or plugging in combination with resurfacing and a preservation of hearing in the majority of patients following transmastoid plugging. Studies showed no subjective worsening after transcanal round window reinforcement. No evidence was available regarding the effect of other techniques. Grade of evidence: IV; grade of recommendation: D.

##### Aural Fullness

Aural fullness was assessed in four studies. Plugging plus resurfacing *via* the middle fossa led to resolution of problems in 28 of the, in total, 33 affected patients and a new development of symptoms in 2/33 patients (19, 20, 30). No evidence was available on plugging or resurfacing *via* the middle fossa. Also no evidence was available regarding transmastoid plugging, resurfacing or a combination of both. Reinforcement of the round window resulted in a significant drop of score for aural fullness on the SCDS 7-point Likert scale (*n* = 22) (7).

Conclusion: Plugging plus resurfacing *via* the middle fossa and round window reinforcement led to a significant reduction in feeling of aural fullness. However, plugging *via* the middle fossa led to the new development of symptoms in two patients. No evidence was available for any of the other techniques. Grade of evidence: IV; grade of recommendation: D.

##### Noise-Induced Vertigo

Improvement of noise-induced vertigo was assessed in seven studies. No evidence was available on plugging *via* the middle fossa. Plugging plus resurfacing *via* the middle fossa resulted in a resolution of symptoms in 49/56 patients (87.5%) and a new development of symptoms in 1 of 56 patients (14, 19, 20, 30). Transmastoid plugging brought resolution in 7/8 patients (10, 12) and transmastoid plugging plus resurfacing led to an improvement in 6/6 patients (25). No evidence was available on transmastoid resurfacing. Round window reinforcement caused a significant reduction in SCDS score from 5.4 to 2.3 on the 7-point Likert scale (*n* = 22) (7).

Conclusion: Noise-induced vertigo improved in the majority of cases following plugging plus resurfacing *via* the middle fossa. No evidence was available regarding plugging or resurfacing *via* the middle fossa. Transmastoid plugging, a combination of plugging and resurfacing as well as round window reinforcement led to a significant reduction in noise-induced vertigo. No evidence was available on transmastoid resurfacing. Grade of evidence: IV; grade of recommendation: D.

##### Pressure-Induced Vertigo

Improvement of pressure-induced vertigo was assessed in 10 studies. No evidence was available regarding plugging *via* the middle fossa. Plugging plus resurfacing *via* the middle fossa resolved symptoms in 45/53 (85%) patients and a new development of symptoms in 1 of 18 patients (14, 19, 20, 30). Resurfacing *via* the middle fossa brought resolution in 12/12 patients (24). Transmastoid plugging led to resolution in 3/6 patients (10, 12) and new development of symptoms in 1/5 (10). Plugging plus resurfacing *via* the same approach led to an improvement but not a resolution of symptoms in all patients (6/6) (25). No evidence was available on transmastoid resurfacing. Round window reinforcement caused a significant decrease in SCDS score for “dizziness with straining” from 2.8 to 1.8 and a significant decrease in score for “ sensitivity to increased middle ear pressure” from 3.6 to 2.0 on the 7-point Likert scale (*n* = 22) (7).

Conclusion: All assessed techniques led to improvement on pressure-induced vertigo in at least 50% of patients. Further comparison of the different techniques was not possible due to difference in outcome measures and a lack of evidence for some techniques. Grade of evidence: IV; grade of recommendation: D.

##### Disequilibrium

Disequilibrium was the most frequently assessed symptom throughout the studies. However, its severity was presented in several different outcome measures and was therefore difficult to compare. Plugging *via* the middle fossa resulted in a decrease of mean DHI from 48.7 (SD 23.4) to 38.2 (SD 30.7, *p* = 0.200) (16). Plugging plus resurfacing *via* the middle fossa resulted in a resolution of symptoms in 38/51 patients and a new development of symptoms in 2 of 51 patients (19, 20, 30). Furthermore, it led to an average decrease in DHI score of 20.45 and 26.0 (*p* < 0.01), respectively (18, 21). One study reported a transient increase in vertigo in the majority of patients (4/5), but to an eventual improvement in all (5/5) patients (22). Resurfacing *via* the middle fossa showed resolution in 12/13 patients (24). Transmastoid plugging was reported to lead to improvements in 9/9 patients (12, 25). However, one larger study reported that it caused transient disequilibrium in 16/16 patients (29). Three studies reported on transmastoid resurfacing. In one study, 29/37 patients felt their disequilibrium had become “much better,” 5/37 felt it was “some better,” 2/37 felt it had remained “the same” while 1/37 reported “worsening” (15). In the other two studies, transmastoid resurfacing was followed by temporary disequilibrium in all cases (4/4 and 3/3) (12, 17). Round window reinforcement caused the SCDS score to significantly drop from 4.2 to 2.1 for general disequilibrium on the 7-point Likert scale (*n* = 22) (7).

Conclusion: Disequilibrium improved or resolved in the majority of patients following all approaches that were assessed. Only transmastoid plugging plus resurfacing was not assessed. However, results were difficult to compare due to the variety of outcome measures. Grade of evidence: IV; grade of recommendation: D.

##### Headache

Improvement of headache was assessed in one study. Plugging plus resurfacing *via* the middle fossa led to a resolution of symptoms in 2/5 patients (*p* = 1.0000) and to a new development of symptoms in 1 of 18 patients. No evidence was available on the other approaches.

Conclusion: Considering the small number of cases assessed it is not yet possible to draw a conclusion regarding the effect of surgery on symptoms of headache. Literature suggests that plugging plus resurfacing *via* the middle fossa can have a beneficial effect in some patients, but can also develop as result of surgery. Grade of evidence: IV; grade of recommendation: D.

##### Quality of Life

The quality of life is often represented by quality of adjusted life years, which can be calculated by different scores such as the HUV. The mean HUV of the general U.S. population is 0.80 (SD 0.29), while SCDS patients (including individuals not undergoing surgery) have a significantly lower mean score of 0.68 (SD 0.13, *p* < 0.01). The change in quality of life for patients undergoing surgery was addressed in 1 of the 19 studies. It reported an increase in HUV from 0.65 (SD 0.12) to 0.79 (SD 0.12, *p* < 0.001) following middle fossa plugging (16). No evidence was available on the other approaches.

Conclusion: Literature suggests that plugging *via* the middle fossa can increase quality of life for patients with SCDS, who generally seem to have a significantly lower HUV in comparison to the general U.S. population. No comparison could be made between the different surgical techniques due to a lack of evidence. Grade of evidence: IV; grade of recommendation: D.

#### Secondary Outcomes—Objective Improvement (Table 4)

**Table 4:** Objective improvement of auditory and vestibular function.

		Study	Results objective measures postoperative relative to preoperative							
			Audiometry	Vestibular-evoked myogenic potentials (VEMPs)	Head impulse test	Calorics	Induced nystagmus	Time	Adverse effects	Hospital stay (days)
Middle Fossa	P	Remenschneider et al. (16) (*n* = 14)	x	x	x	x	x	*Health utility value, autophony index (AI), dizziness handicap inventory (DHI), Hearing Handicap Inventory*: 3 months	x	x
	
	P + R	Chung et al. (30); 21 ears (*n* = 18)	x	x	x	x	x	x	x	x
		
		Thomeer et al. (19); 16 ears (*n* = 13)	*0.25–1 Hz (n *=* 16)*: Air conduction (AC): mean ↑: +7.4 dB (SD 7.7) Bone conduction (BC): mean ↓: −2.5 dB (SD 7.2) Air bone gap (ABG): mean ↓: −9.0 dB (SD 10.9) *0.5–4 Hz*: AC: mean ↑: +4.5 dB (SD 5.3) BC: mean ↓: −0.2 dB (SD 5.6) ABG: mean ↓: −4.7 dB (SD 6.9) *Speech Reception Threshold*: 7 days: +11.6−dB 1–6 months: −2.2 dB *p* > 0.05 *Sensorineural hearing loss (SNHL)*: 3/16	Cervical vestibular-evoked myogenic potential (cVEMP) Thresholds (n = 14) Pre Mean: 76.1 dB (range 70–90) (abnormal <90 dB nHL) Post mean: 94.4 dB (range 80–100) (*p* < 0.0021) *Amplitudes*: normalized (*p* = 0.34) Not further defined	x	x	x	x	x	7.8 (range 4–13) ICU: 2.7 (2–8)
		
		Goddard and Wilkinson (20); 24 ears(*n* = 23)	0.5–3 kHz AC (*n* = 23): Pre mean pure-tone average (PTA): 21.1 dB (SD 21.6) Post mean PTA: 22.5 dB (SD 16.7) *p* = 0.42 BC (*n* = 16): Pre mean PTA: 15.6 dB (SD 14.1). Post mean PTA: 16.2 dB (SD 17.1) *p* = 0.81 ABG (*n* = 14): mean change: +1.7 dB (not significant) *Word Recognition Score (n *=* 23)*: Pre: 95.8% Post: 95.1% *p* = 0.48	x	x	x	x	x	Tegmen mastoideum Defect: 4/24 Temporary facial weakness: 1/24	x
		
		Ward et al. (34); 43 ears (*n*=40)	0.25–8 kHz (n *=* 43) *AC*: 0.25–1 kHz: no change 2–8 kHz: significant increasein mean thresholds 0.25–4 kHz (n *=* 43) BC: Pre Mean PTA: 8.4 dB (SD 10.4) 7–10 days: 19.2 dB (SD 10.4)*p* < 0.05 >1 month: 16.4 dB (SD 18.8) *p*=0.01 0.25–2 kHz (n*=*43) ABG: Pre: 16.0 dB (SD 7.6) 7–10 days: 16.4 dB (SD 11.1) >1 months: 8.1 dB (SD 8.4) *p* < 0.05 Speech Discrimination Score, % (SD) (n*=*43) Pre: 98.6 (SD 4.1) 7–10 days: 94.4 (SD 14.5) >1 months: 93.1 (SD 19.6) *SNHL*: transient >50%, persistent: 25%.	x	x	x	x	x	Hemotympanum/middle ear effusion (7–10 days post): 84% Subgroup had significantly larger ABG: 18.1 dB (SD 11.4) vs. 8.8 dB (SD 3.9) *p* < 0.05	x
		
		Agrawal et al. (26); 42 ears	x	x	VOR gain Horizontal: >0.7 OR clinically normal: Pre: 18/18 1 week post: 11/18 |>6 weeks post: 16/18	x	x	x	x	x
		Crane et al. (18); 19 ears	*0.25–8 kHz AC*: not reported *0.25–4 kHz BC*: not reported ABG (largest): Pre: 25.83 dB (usually 0.25 kHz) Post: 15.55 dB (frequency not reported) Hearing (n *=* 18) Decreased: 11/18 Increased: 3/18 No change: 4/18	cVEMP *Thresholds (n =#x000A0;8*) Pre mean: 67.5 dB Post mean: 69.1 (absent in 2) Increased: 5/6, unchanged: 1/6 Normal: >80 dB nHL	x	x	x	*AI, DHI*: 3 months	x	x
		
		Crane et al. (21); 19 ears	0.25–8 kHz (n *=* 18) AC: mean ↑: 10 ± 23 dB (range −45 to +45 dB) 0.25–4 kHz BC: mean: not reported	cVEMP *Thresholds*: Pre (*n* = 19): mean: 73 ± 8 dB (range 60–90) Threshold <85 dB: 16/19 Post (*n* = 11): threshold >85 dB OR Absent: 10/11	x	x	x	*DHI*: 3 months	x	x
		Carey et al. (14); 19 ears	x	x	VOR gain (n *=* 19) Horizontal: Pre: 0.94 ± 0.07 Post: 0.90 ± 0.24 % Change −5% (*p* = 0.33) Superior: Pre: 0.75 ± 0.13 Post: 0.42 ± 0.11 % Change: −44% (*p* < 0.0001) Posterior: Pre: 0.84 ± 0.09 Post: 0.73 ± 0.20 % Change: −13% (*p* = 0.018)	x	x	x	Epidural hematoma: 1/19 Cellulitis of the wound: 1/19 Transient diabetes insipidus: 1/19	x
		
		Phillips et al. (22); 5 ears	(Frequencies not reported) (n *=* 5) AC, BC: correction of pseudo-conductive hearing loss: 4/5 *SNHL*: temporary: 3/5 Residual (>4 months): none	cVEMP *Thresholds (n = 5)*: Pre: 65 dB (60–70) Post: 82 dB (75–90) Normalized: 5/5	x	x	x	4 months	Vertigo: Transient: 4/5 Persistent: 1/5	x
		
		Limb et al. (23); 29 ears	0.25–8 kHz (n *=* 29) AC: no sign.change 0.5–4 kHz BC: no sign.change 0.25–8 kHz (n *=* 29) ABG: partial closure: 5/29 *SNHL*: association with positivesurgical history *Speech Discrimination Score* per subgroup: 1. No surgical history (n *=* 19) Pre: 96.5 ± 5.0% Post: 96.8 ± 3.8% (*p* = 0.66) 2. Prev. MEE/PE tube (n *=* 6) Pre: 98 ± 2.3% Post: 99 ± 2.1% (*p*: not reported) 3. Prev. stapes procedures (n *=* 3) Pre: 98.7 ± 2.3 Post: 65 ± 56.6%(*p*: not reported)	x	x	x	x	x	x	x
	
	R	Hillman et al. (24); 16 ears (*n* = 13)	0.5–3 kHz (n *=* 16) AC, BC not reported ABG >10 *(freq. not reported)* Pre: 2/16 Post: 2/2 decreased *SNHL*1/16	x	x	x	x	>3 months	Reoccurrence of symptoms after 5 months due to a shift of the bone cement: 1/14	x

Transmastoid	P	Van Haesendonck et al. (10); 12 ears	0.5–4 kHz (n *=* 12) AC: Pre median PTA: 25 dB post median PTA: 18 dB Median change: 1 dB BC PTA: Pre median PTA: 11 dB Post: 16 dB Median change: −4 dB ABG Pre: 13 dB Post: 5 dB(median change:8 dB) *SNHL*: not observed	x	x	x	x	1–6 months	Posterior canal BPPV: 2/12 (solved through Epley maneuver)	x
		
		Beyea et al. (29); 16 ears	x	x	x	x	x	x	Temporary vertigo: 16/16 Dural tear: 2/16 (low-lying tegmen both cases)	x
		
		Agrawal and Parnes (12); 3 ears	0.25–8 kHz (n *=* 3) AC, BC: not reported ABG: decreased: 1/2 *Hearing*: preserved: 3/3, *|Word Recognition Score*: preserved: 3/3		x	x	x	x	Temporary vertigo: 3/3 Dural tear: 1/3	x
	
	P + R	Fiorino et al. (25); 6 ears	(Freq. not reported) (n *=* 6) AC, BC: PTA change <10 dB: 4/6 Conductive hearing loss: Unchanged: 1/1 Newly developed due to MEE: 1/6	VEMP Thresholds (n = 6) Pre Mean: 74 dB (1 absent) Post: >90 dB nHL: 5/6 Stayed absent: 1/6	x	Procedure not described (*n* = 6) Reduced caloric response: 2/6	Procedure not described (*n* = 6) Present: 0/6	1.5–2 months	Pneumonia: 2/6	2–5
	
	R	Amoodi et al. (17); 4 ears	0.25–8 kHz (n *=* 4) *AC, BC*: general improvement: 3/4 *AC*: improvement: 4/4 *BC*: unchanged: 4/4 *ABG*: partial/complete losure: 4/4	x	x	x	Procedure not described (*n* = 4) Present: 0/4	Not reported. Follow-up: 1.5–4 years	Temporary vertigo: 4/4	<1
		
		Lundy et al. (15); 37 ears	(Freq. not reported) (n *=* 37) AC, BC: not reported *SNHL*: 2/37 (dehiscence >5 mm)	x	x	x	x	>3 months	x	<12 h: 13/37 24 h: 24/37

Transcanal	RW	Silverstein et al. (7); 19 ears	x	x	x	x	x	x	x	

##### Audiometric Findings

###### Pure-Tone Audiometry: AC, BC, ABG, and Sensorineural Hearing Loss (SNHL)

Among all studies, 13 reported their audiometric outcomes. Due to the difference in outcome measures, it was, however, not feasible to pool the results (Table 2).

Following plugging plus resurfacing *via* the middle fossa, one study found an improvement of AC and a decrease in BC, resulting in an at least partial ABG closure on all frequencies (0.25–4 kHz) in the majority of cases within the study. SNHL was reported in a minority of cases (3/16) (19). Another study did not report thresholds, but described a general improvement of AC with an average of 10 ± 23 dB HL (21). In contrast, a large-series study found no change over the lower frequencies and a significant decrease in AC and increase in BC for the higher frequencies (2–8 kHz). It showed an acute high-frequency SNHL in >50% of patients that persisted in 25% of them (13).

Another study reported a decrease in hearing in the majority of patients (11/18), despite a reduction in mean of the largest ABGs. This effect is most likely due to an overall decrease in BC and not an increase in AC (18). The study further reported no change in hearing in 4/18 patients, and improvement in the minority of patients (3/18) (18).

One large-series study found no significant change in AC, BC, and ABG over the frequencies 0.5–3 kHz. It did not address higher frequencies (20). Another study reported no significant AC or BC changes after surgery in the general SCDS population. The study also found a partial ABG closure of the ABG in a minority of patients (2/29). However, in some cases SNHL did occur. It was associated with a positive previous history for stapes surgeries (23). One other smaller study did not report AC and BC values, but described a correction of pseudo-conductive hearing loss in the majority of patients (4/5) with only temporary SNHL loss in 3/5 patients (22).

Resurfacing the dehiscence *via* the middle fossa was described in one study, which did not report AC and BC values, but described ABG closure in all affected patients with a preoperative ABG >10 dB (2/2) and SNHL in only 1 out of 16 cases (24).

Following transmastoid plugging one study found decrease in median AC PTA from 25 to 18 dB and an increase in median BC PTA from 11 to 16 dB. The ABG shrunk from 13 to 5 dB and no SNHL was observed (10). Two studies did not report audiometric data, but reported general results. One showed that transmastoid plugging resulted mainly in “preservation” (14/16) of hearing and in an “improvement” in 2/16 patients (29), and one study reported a decrease in ABG in 1/2 patients, “preservation” in hearing in 3/3 patients (12).

Plugging plus resurfacing led to a change of PTA <10 dB in 4/6 patients, meaning there was no significant change in these patients. It was not described whether PTAs increased or decreased (25). Conductive hearing loss remained unchanged in one affected patient (1/1) and newly developed due to middle ear effusion in 1/6 patients (25).

Transmastoid resurfacing led to an AC improvement (4/4) and no BC (4/4) in one study. The study reported at least partial closure of the ABG in all patients (4/4) (17). Another study did not announce audiometric data, but reported that SNHL was found in 2/37 patients (15). Of these two patients, one was congenitally deaf. She had a small amount of residual hearing in the involved ear, which she lost postoperatively. The second patient had a drop in sensorineural levels from normal to 40 to 75 dB bone threshold. Both had a relatively large dehiscence of 5 mm.

Conclusion: The way of assessing the results on AC, BC and ABG varied significantly among the studies. No conclusion could be drawn regarding the effect of plugging plus resurfacing *via* the middle fossa on hearing outcomes. However, some existing literature suggests that it might cause high-frequency SNHL (13). Resurfacing *via* the middle fossa led to closure of the ABG in all affected cases and caused SNHL in only 1 of 16 cases (24). Transmastoid plugging, resurfacing and the combination of both seem to be a generally safe technique regarding hearing outcomes. Transmastoid resurfacing led to SNHL in very few cases (2/41) (15, 17). However, in most patients the range of frequencies is not reported, which means that a high-frequency loss cannot entirely be ruled out. No assessment was done following round window reinforcement. Grade of evidence: IV; grade of recommendation: D.

###### Speech Audiometry

Speech audiometry was performed in very few studies and also assessed using different outcome measures, which did not allow pooling of the results. Outcome measures included: the SRT (19), the Word Recognition Score (20), and the Speech Discrimination Score (13, 23).

The SRT increased in the first week after surgery and decreased again under the baseline after 1–6 months (19), which demonstrated an only temporary hearing loss following plugging plus resurfacing *via* the middle fossa. The WRS remained unchanged following plugging plus resurfacing *via* the middle fossa (23/23 patients) (20) and transmastoid plugging (3/3 patients) (12). The Speech Discrimination Score decreased significantly following plugging plus resurfacing *via* the middle fossa in the first 7–10 days and decreased further >1 month postoperatively (*n* = 43) (13).

Conclusion: Speech audiometry is performed in very few studies and the outcome measures vary. However, the assessment of speech audiometry is often part of the publication criteria when reporting hearing outcomes (31). It is therefore desirable that future reports on hearing outcome contain a standardized assessment of speech audiometry. Grade of evidence: IV; grade of recommendation: D.

##### Vestibular Findings

###### Cervical and Ocular Vestibular-Evoked Myogenic Potential (cVEMP/oVEMP)

Vestibular-evoked myogenic potentials were assessed in five studies of which four assessed cVEMPs and one did not announce if it concerned cVEMPs or oVEMPs (25). Thresholds were reported in the majority of studies, while amplitudes were only described in one study. Regarding the thresholds, the reference values varied per laboratory between “normal >80” (18) “normal >85 dB” (21), and “normal >90 dB” (19, 25).

Three studies assessed results after plugging plus resurfacing *via* the middle fossa. All three studies described an increase in mean VEMP threshold in the majority of patients (18, 19, 21). However, in one study, the mean threshold remained <70 dB (18), in one it increased to >80 dB (21), and in one study it increased >90 dB in all affected patients (19). In the latter study, amplitudes “normalized” after plugging plus resurfacing; however, reference values were not further defined (19). Another study reported that VEMP thresholds normalized in all cases (5/5), with the mean postoperative thresholds being 82 dB. Reference values were not defined (22).

Regarding the transmastoid approach, VEMPs were studied only in one study where plugging plus resurfacing was performed (25). A normalization of VEMP thresholds >90 dB in 5/6 cases was reported. In one case, the threshold remained absent (25).

Conclusion: Generally when the change in VEMP threshold was analyzed, an increase of the threshold was reported. However, one study did not report the exact type of VEMP that was assessed and a number of studies did not report VEMP thresholds at all.

Plugging plus resurfacing the canal *via* the middle fossa seems to lead to an increase of VEMP thresholds in the majority of cases. No literature was available on the effects of resurfacing *via* the middle fossa and transmastoid plugging or resurfacing.

Little literature was available on the effect of transmastoid plugging plus resurfacing, but the existing studies showed that it leads to an increase in VEMP threshold >90 dB in most cases.

No assessment was done following round window reinforcement. Furthermore, it is important to recognize that reference values for VEMP thresholds differ vastly between laboratories. Therefore, consensus is needed how to report on VEMPs. Grade of evidence: IV; grade of recommendation: D.

###### HIT and vHIT (HIT/vHIT)

Only two studies assessed the effect of surgery on the HIT. One other study mentioned HIT results for only two patients. No further pre- and postoperative data of the remaining population were reported (25). In both cases, the assessment followed plugging plus resurfacing *via* the middle fossa. One study assessed the horizontal canal VOR gain quantitatively (using vHIT) in the preoperative phase, qualitatively (using HIT) in the initial postoperative period (after 1 week) and qualitatively and quantitatively at more than 6 weeks postoperatively (26). It was found that the number of patients with a VOR gain ≥ 0.70 dropped from 18/18 preoperatively to 11/18 one week after surgery, but then increased again to 16/18 six weeks postoperatively (26) implying a transient loss of horizontal canal function.

The other study assessed the VOR gain for all canals using vHIT. A significant decrease in gain of 44% (*p* < 0.0001) was found for the superior canal. The gain for the ipsilateral posterior canal dropped by 13% (*p* = 0.018), while the average gain for the ipsilateral horizontal canal did not change significantly (*p* = 0.33) (14). No assessment was performed following the other approaches.

Conclusion: The evidence was not sufficient to compare the effect of the different surgical techniques on VOR gain. However, literature suggests that plugging plus resurfacing the dehiscence *via* the middle fossa leads to a significant decrease in VOR gain and therefore to a hypofunction of the superior canal. Furthermore, it can lead to temporary loss of function of the horizontal canal. Grade of evidence: IV; grade of recommendation: D.

###### Caloric Response

No literature was available on the change in caloric response following plugging, plugging plus resurfacing or resurfacing *via* the middle fossa. Caloric response was assessed in only one study (25), which analyzed the response following transmastoid plugging plus resurfacing. The study reported a reduced caloric response in 2/6 patients. Both cases had expressed a reduced response preoperatively. No literature was available analyzing caloric response following transmastoid resurfacing or plugging alone. No assessment was done following round window reinforcement.

Conclusion: The evidence for the effect of surgery on caloric response was not sufficient enough to draw conclusions, but existing literature suggests that there is no decrease in caloric response following transmastoid plugging plus resurfacing. Grade of evidence: IV; grade of recommendation: D.

###### Induced Nystagmus

No literature was available on the presence of induced nystagmus following plugging, plugging plus resurfacing or resurfacing *via* the middle fossa. It was assessed in only one study that analyzed the presence of nystagmus following transmastoid plugging plus resurfacing. Preoperatively sound-, pressure-, and Valsalva-induced nystagmus was seen in 5/6, 4/6, and 5/6 patients, respectively. No induced nystagmus was observed postoperatively (25). No literature was available that analyzed induced nystagmus following transmastoid resurfacing or plugging. Also, no assessment was done following round window reinforcement.

Conclusion: The evidence for the effect of surgery on the presence of induced nystagmus was not sufficient enough to compare the different surgical techniques, since only transmastoid plugging plus resurfacing was investigated. Grade of evidence: IV; grade of recommendation: D.

##### Adverse Effects

The presence of adverse effects was assessed in 11 studies. No evidence was available on the effects of plugging *via* the middle fossa. Studies that looked at adverse effects after plugging plus resurfacing *via* the middle fossa reported the following: facial weakness (1/24), dural tears (4/24) (20), hemotympanum and middle ear effusions associated with a temporarily increased ABG postoperatively (37/43) (13), epidural hematoma (1/19) (14), cellulitis of the wound (1/19) (14), and temporary diabetes insipidus (1/19) (14).

Resurfacing *via* the middle fossa led to a recurrence of symptoms after five months due to a shift of bone cement in one case (1/14) (24).

Transmastoid plugging caused posterior canal BPPV in 2/12 patients (10). Furthermore, a larger study reported dural tears in 2/16 patients. Both patients had evidence of a low-lying tegmen (29).

Following plugging plus resurfacing, 2/6 patients developed pneumonia (25).

Transmastoid resurfacing was complicated by a dural tear in 1/3 cases (12). No evidence regarding adverse effects following round window reinforcement *via* the ear canal was available.

Conclusion: The severity of local adverse effects was higher following the middle fossa approach than the transmastoid approach. A transient increase in vertigo seemed to develop in the majority of cases regardless of the approach. The risk for intraoperative dural tears using the transmastoid approach seemed to be higher in patients who had evidence of a low-lying tegmen. Grade of evidence: IV; grade of recommendation: D.

##### Length of Hospital Stay

The length of hospital stay was reported in four studies. No evidence was available on the effects of plugging *via* the middle fossa. Following plugging plus resurfacing *via* the middle fossa the average hospital stay was 7.8 days (range 4–13), while the ICU stay was 2.7 days (range 2–8) (19). The exact reason why patients were admitted to the ICU was not reported. No evidence was available following resurfacing alone *via* the middle fossa. Also no evidence was available following transmastoid resurfacing.

Transmastoid plugging plus resurfacing was followed by an average hospital stay of 2–5 days (25). Transmastoid resurfacing showed the shortest hospital stays. One study reported an average hospital stay of <24 h (17), and another study reported that 13/37 patients were released on the day of surgery and 24/37 had one overnight stay but were also released within 24 h (15).

Conclusion: According to the available evidence, plugging plus resurfacing *via* the middle fossa resulted in a longer hospital stay than transmastoid plugging plus resurfacing or transmastoid resurfacing alone. Furthermore, ICU admission was reported in some cases following plugging plus resurfacing *via* the middle fossa, although it is unclear if this was performed routinely or triggered by a specific indication. Transmastoid resurfacing presented with the shortest hospital stays. This information has to be interpreted with great care since significant differences in health care organization may also explain hospital stay. Grade of evidence: IV; grade of recommendation: D.

## Discussion

This literature review highlights difficulties that researchers face when investigating a relatively new condition. The majority of included studies were retrospective and since SCDS is rare, studies inevitably have small sample sizes. Furthermore, the lack of standardized outcome measures made it difficult to pool or compare outcomes (Table 2). Comparison was difficult due to the following factors: anamnesis was mostly performed in a non-standardized manner, which led to several different subjective (primary) outcome measures. Furthermore, several objective (secondary) outcome measures like audiometry, VEMPs, HIT, calorics, and presence of induced nystagmus were assessed in different ways. Therefore, most results from the different studies, regarding primary as well as secondary outcome measures, could only be summarized qualitatively and could not be statistically pooled.

Even though a number of significant reviews have been published since the last database search of this review, the majority of them did not address this particular issue (32, 33).

Some findings, however, were consistently reported. Overall an improvement of clinical symptoms and objective measures was found in the majority of patients regardless of the approach or closure technique. In most cases adverse effects or worsening of vertigo or hearing were temporary, typically resolving after several weeks. The surgical approach appears to determine the length of hospital stay, although differences in health care organization need to be considered. On average the middle fossa approach was followed by a longer hospital stay than the transmastoid and transcanal approaches. Adverse effects varied depending on the type of approach and anatomical features of the patient.

From the literature reviewed, it appears that surgery has a positive impact on the symptoms of SCDS but due to the use of different non-standardized outcome measures, surgical techniques could not reliably be compared. This is consistent with recent findings (34). Agreed upon standardized assessment tools, outcome measures and set timeframes are needed.

It is important to mention that not all the available diagnostic tools were addressed within this literature review, as some of them were not applied in any of the eligible studies. For example, it has previously been reported that the air conducted ocular VEMPs are superior to cVEMPs in the diagnosis of SCDS, and that electrocochleography could reflect the presence of a third mobile window (35–37). Future SCDS studies could therefore take these other diagnostic tools into consideration.

For a standardized protocol we strongly recommend the inclusion of assessments of symptom severity and changes in vestibular as well as auditory function in patients with SCDS before and after surgery at set times.

### Limitations

#### Review Level

Although this review aimed to evaluate the available literature in a systematic fashion, the literature search was done by the first author alone. The evaluation was done by the first and last author. Furthermore, not all available databases have been searched and the reviewing process was restricted only to published reports. No correspondence was established with the authors of the individual studies to obtain unpublished data. Therefore, publication bias might have influenced the conclusion of this review.

#### Study Level

On the study level, this review is limited by the retrospective nature of the majority of the analyzed studies. All reviewed studies show relatively weak internal validity due to small populations, high risk for both selection and information bias. The prospective study presented in this review consisted of a larger population, but was neither blinded nor randomized. Therefore, internal validity of this study was also moderate. The differences in methodology among studies made a comparison and pooling of the results impractical.

### Conclusion

Generally, surgery has a positive impact on the symptoms of SCDS but due to the use of different outcome measures among studies, surgical techniques could not be compared quantitatively. Standardized assessment tools, outcome measures and set timeframes for patient follow-up are needed. Based on this review, we recommend the inclusion of assessments of symptom severity and changes in both vestibular as well as auditory function in patients with SCDS before and after surgery into the standardized protocol.

## Author Contributions

MO: study design, literature search, data analysis, and writing of the article. RS: study design and reviewing manuscript. HK, JT, VR, and YT: reviewing manuscript. RB: study design, data analysis, and reviewing manuscript.

## Conflict of Interest Statement

The authors declare that the research was conducted in the absence of any commercial or financial relationships that could be construed as a potential conflict of interest.
